# Comparison of patient perceptions of Telehealth-supported and specialist nursing interventions for early stage COPD: a qualitative study

**DOI:** 10.1186/s12913-016-1623-z

**Published:** 2016-08-22

**Authors:** Deborah A. Fitzsimmons, Jill Thompson, Claire L. Bentley, Gail A. Mountain

**Affiliations:** 1 School of Health Studies, Western University, Arthur and Sonia Labatt Health Sciences Building, London, Ontario N6A 3B4 Canada; 2 School of Health and Related Research, The University of Sheffield, Regent Court, 30 Regent Street, Sheffield, S1 4DA UK; 3 School of Nursing and Midwifery, The University of Sheffield, Barber House Annexe, 3a Clarkehouse Road, Sheffield, S10 2LA UK

**Keywords:** Qualitative, Patient-centred care, Innovation, Technology, Telehealth, COPD

## Abstract

**Background:**

The increasing prevalence and associated cost of treating Chronic Obstructive Pulmonary Disease (COPD) is unsustainable, and focus is needed on self-management and prevention of hospital admissions. Telehealth monitoring of patients’ vital signs allows clinicians to prioritise their workload and enables patients to take more responsibility for their health. This paper reports the results of a qualitative study embedded within a feasibility and pilot Randomised Controlled Trial (RCT) of Telehealth-supported care within a community-based COPD supported-discharge service. The aim of the study was to qualitatively explore the experiences of patients with COPD who had received either a Telehealth-supported or a specialist nursing intervention following their discharge from hospital after an admission for a COPD exacerbation.

**Methods:**

Patients were invited to either participate in semi-structured interviews or to complete a semi-structured self-administered questionnaire on completion of the intervention. Nine patients were interviewed (67 % female) and seventeen patients completed the questionnaires. In addition, three clinicians responsible for the delivery of both interventions were interviewed to obtain their perspectives on the new services.

**Results:**

Seven underlying themes emerged from the patient interviews and were further explored in the questionnaires: (1) patient demographics; (2) information received by the participants; (3) installation of the Telehealth technology; (4) Telehealth service functionality; (5) visits; (6) service withdrawal; and (7) service perceptions. Recipients of both services reported feelings of safety derived from the delivery of an integrated, community-based service.

**Conclusions:**

Although recipients of the Telehealth service received 50 % fewer home visits from the clinicians than recipients of a more traditional community-based nursing intervention, the patients were enthusiastic about the service, with some describing it as the best service they had ever received. This suggests that a Telehealth intervention is an acceptable alternative to a more traditional home nursing visit model for monitoring community-based patients with COPD following their discharge from hospital.

**Trial registration:**

Current Controlled Trials ISRCTN68856013

**Electronic supplementary material:**

The online version of this article (doi:10.1186/s12913-016-1623-z) contains supplementary material, which is available to authorized users.

## Background

Existing models of health service provision for patients with long term conditions are unsustainable and patients need to be supported to manage their disease [1], thereby avoiding hospital admission [2]. One such long term condition is Chronic Obstructive Pulmonary Disease (COPD), a prevalent disease [1] characterised by progressive worsening of lung capacity, frequent hospital admissions, high levels of disability, and depression [3–5].

Telehealth monitoring is defined as the remote exchange of physiological data between a patient at home and medical staff to assist in diagnosis and monitoring. It includes (amongst other things) a home unit to measure and monitor temperature, blood pressure or other vital signs for clinical review at a remote location (for example, a hospital site) using phone lines or wireless technology [6]. The use of community based medical monitoring technology offers real potential for people with long term conditions in that it removes the physical location aspect of health care. Consequently it is believed that effective use of Telehealth could lead to significant cost savings [7].

Whilst a recent Cochrane review [5] demonstrated the potential for Telehealth monitoring in reducing hospital admissions and increasing quality of life in people with a clinical diagnosis of COPD, the review found that Telehealth was usually delivered as part of a more complex package of care, making it difficult to separate the effects of the technology from other aspects of the service. A recent, large scale UK trial of Telehealth demonstrated that this technology has the potential to reduce mortality and emergency admission rates [8]. However, studies have failed to demonstrate cost-effectiveness of the technology so far [8]. Implementation of Telehealth for the management of long term conditions remains a policy priority in the UK [9] and internationally [10, 11].

The term ‘Telehealth’ has been used to describe many forms of digital service provision enabled by information and communications technology. However, ‘Telehealth’ can be differentiated into digital tools, such as the use of video- conferencing to reduce the need for face-to-face visits [12–16] (defined by the NHS Commissioning Assembly as telemedicine or teleconsultation) [17] or telephone interventions to support people by building knowledge, skills and confidence to change behaviours [18] (defined by the NHS Commissioning Assembly as telecoaching) [17]; and the use of technology to enable health service delivery, such as the remote monitoring of patients in their own homes to “anticipate exacerbations early and build their self-care competencies” [17]. Telehealth monitoring interventions differ widely, using a range of devices, including web-phones with touchscreens used to enter patient data [19], mobile phones with peripheral devices [20], or home units like the one described in this study, used with a variety of peripheral devices [21, 22], Even where comparable technology has been used, studies are for different clinical populations [23, 24], who may have previously received different clinical interventions; [25] collect different physiological data; [26, 27] or the interventions were of significantly different durations [25], so the findings may not be transferrable.

Given the diversity of these technology-enabled systems, it is not surprising that one Cochrane review [28] has identified the need for additional qualitative research to determine why particular telehealth interventions are, or are not, successful. Another systematic review [29] identified that few studies to date have reported on patient satisfaction with telehealth-enabled services. Larrabee et al. [30] suggest that “many patient satisfaction instruments are not based on patient perceptions, theoretically limiting their validity”. Demeris et al. [13], go further and suggest that a focus upon patient satisfaction following receipt of a Telehealth intervention fails to “identify the attributes of the system evaluated as satisfactory, or more importantly, the general perception patients have of the system”. Larrabee et al. [30] identified five themes associated with patient perceptions of high quality nursing care: providing for my needs, treating me pleasantly, caring about me, being competent, and providing prompt care. Whilst a satisfaction survey should consider whether all of these themes are addressed and the degree to which this is achieved, it is by considering *how* these themes are addressed that the successful, or the unsuccessful, attributes of the service may be fully understood. It is this level of qualitative analysis that is still missing from the evaluation of Telehealth interventions [28].

Consequently, this study was designed to go beyond the satisfaction assessment of Telehealth interventions and qualitatively explore the experiences of patients with COPD who had received either an eight week Telehealth monitoring or a nursing intervention for ‘early stage’ COPD following their discharge from hospital in order to identify what they consider to be limitations or contributors to the effectiveness of these specific interventions, and to examine where patient’s perceptions matched or differed between the two service delivery modalities.

### Local context

The research described in this paper was undertaken in collaboration with one Primary Care Trust (PCT). Created in 1999 [31], PCTs were statutory bodies in the publicly funded English National Health Service (NHS). Prior to their replacement with Care Commissioning Groups (CCGs) in 2013, the PCT in question was one of 152 such organisations in England [32]. Budgets for PCTs were determined by the Department of Health and they were responsible for over £80 Billion or over 80 % of expenditure on the National Health Service [32]. Managed by a board of directors, including non-executive directors selected following open recruitment, and lead by a Chief Executive, the PCTs had two distinct roles: the commissioning of primary, community and secondary health care services from providers for their local population; and direct delivery of community-based health care services [33]. Following the abolition of the PCT, members of the commissioning team moved to the CCG for the region, and the health service delivery unit was transferred to an NHS foundation trust providing a range of community, mental health and learning disability services across a broader geographical area [34], thereby creating continuity of service delivery. Responsible for a local population of over 230,000 [35], the PCT had a disproportionate number of individuals over the age of 65 in the community and a projected increase of 67 % in the over 65 age group by 2031 [36], posing a local health care planning challenge. Additionally the population served by the PCT had a high incidence of COPD due to its coal mining history, and high levels of deprivation and poor lifestyle [37–39], To respond to the needs of its population, the PCT commissioned a specialist COPD post discharge service for up to eight weeks for people who had exacerbation-related hospital admissions. The discharge service employed two COPD Specialist Nurses, one Specialist Physiotherapist and one Community Matron to treat patients meeting the referral criteria (shown in Table 1).

**Table 1:** Eligibility criteria for acceptance into the COPD discharge service

SpO_2_ levels	>90 % on air or pO_2_ > 7 kPa/pH 7.35–7.45
Respiratory rate	<25
Temperature	<37.8 °C
Systolic blood pressure	90–180 mm/Hg
Pulse	50–100 BPM
Status	Orientated and alert/able to give consent
Environment	Safe discharge environment
COPD stage	Fewer than three hospital admissions (including the current admission) during the prior twelve months from the date of discharge with COPD as the documented reason for hospital admission

The service was designed to assist patients to manage their illness more effectively with the aim of decreasing readmission rates. Patients received six home-based visits from one of the discharge service team over eight weeks post discharge from acute care. This model of care was perceived to be highly successful and the PCT was consequently instructed to increase the remit of the service to all patients discharged from acute care with COPD but not necessarily having been admitted to hospital with an exacerbation. They also had to do so with minimal increase in resources.

To achieve the expansion of the service from 60 to potentially 1200 patients per year using the previously described service delivery model of face to face patient contacts in the home was not feasible. A more efficient solution was required which could only be achieved through a radical change in care delivery methods. Consequently, the PCT decided to implement a Telehealth supported discharge service which, like the standard nursing service, would be provided to the patient without charge at the point of care.

The PCT introduced the ‘Telehealth service’ to deliver care that would be acceptable to patients; decrease hospitalisations; improve the quality of life for patients; and also to reduce the use of resources by people with COPD while at the same time significantly increasing the patient numbers receiving the service. It was believed that the data collected through the Telehealth system would enable clinicians to identify whether patients required additional supportive home visits to address any fluctuations in their condition and would reduce unnecessary visits, thereby freeing resources to support additional patients whilst still providing patient-centred care. The two care pathways for the original service (standard service) and the Telehealth supported service are shown in Table 2.

**Table 2:** Standard and Telehealth interventions

	Time Line	Standard Service Care Pathway	Telehealth Service Care Pathway
Intervention	Day 1 < 24 hours after hospital discharge	Home visit	Home visit
	Day 3	Home visit	Home visit
	Day 5	Home visit	Telehealth equipment installed
	Week 2	Home visit	Remote review of Telehealth parameters throughout intervention
	Week 6	Home visit	
	Week 8	Discharge home visit	Discharge home visit Telehealth equipment removed

The selected Telehealth system (Doc@Home®) provided both monitoring and self-management support functionality. Using a small hand-held device, patients were required to answer tailored questions about their health status by reading questions on the screen of the device and pressing the appropriate response button. Patients also used a blood pressure monitor and oximeter peripherals to measure their blood oxygen levels each day. The peripherals were connected by Bluetooth to the hand-held device, and all readings were transmitted to a secure web-based server by telephone line, ready for access by the clinicians. Patients were able to observe their readings each day, and this was a core educational element of the service. The system generated an alert if reported signs and symptoms fell outside clinician-generated thresholds, or if the patient failed to undertake monitoring activity. To ensure comparability with the standard service [40], equipment was provided at home to those receiving the Telehealth supported service for eight weeks.

Installation of the Telehealth equipment involved the installer instructing patients (and if appropriate their carer) on how to use the equipment (including the peripherals). The installer also informed them of when they should take readings as well as how and when they might request help if required. The installer provided the patient with a customised instruction manual which included service information and key contact details should they require assistance.

The study [41], which involved the collection of patient perceptions of the Telehealth technology, included a feasibility and pilot randomised controlled trial in preparation for a fully powered trial. Participants were recruited from those referred to the discharge service who were willing to accept Telehealth as part of their discharge plan; were able to communicate in, and read English (a requirement of the technology); had a telephone landline in their home; were free of any cognitive or other impairment that could impede ability to participate; were free of comorbidities requiring ongoing intervention from other community services; and were not identified by their GP as being unsuitable to participate. Patients failing to meet the inclusion criteria and in particular those who were not able or willing to use Telehealth were offered the standard service. Of 450 patients assessed for pilot study eligibility, 180 were excluded as they did not meet the inclusion criteria.

Two referral routes into the PCT COPD service from the hospital were identified:

1. The hospital employed two specialist COPD nurses who could refer people directly to the Community COPD Service for early discharge from their care; alternatively
2. Any member of the hospital nursing staff could use a telephone referral service for patients with a diagnosis of COPD (which may be in addition to other conditions) who were being discharged from the hospital.

Additionally, family physicians, family members and patients themselves were able to contact the Community COPD Service directly. The administrator for the discharge service triaged any patients referred though routes other than the hospital COPD nurses to ensure that they were eligible to use the service.

## Methods

### Overview

This qualitative study was embedded within a feasibility study and pilot trial [39] completed in preparation for a future randomized controlled trial of Telehealth monitoring for patients with early stage COPD. [41] The feasibility study was conducted over a period of 5 months and the pilot RCT over 14 months [40]. The semi-structured interviews were undertaken to help the researchers understand the patient service experience.

### Patient interviews

During the feasibility study, semi-structured interviews were conducted with former recipients of both the Standard Service and the Telehealth Service. PCT clinicians identified patients who were due to be discharged from their caseload for either the standard Service or Telehealth service. For details of the recruitment and consent processes please refer to the ‘ethics approval and consent to participate’ information in the disclosures.

Nine patient interviews were undertaken: five with individuals who had completed the Telehealth Service and four with recipients of the standard service. A topic guide for the interviews [see Additional file 1] was devised based on the literature. Prior to submitting the topic guide for approval to the NHS ethics committee, the guide was reviewed by the research advisory group for the study to determine whether the aims of the study would be addressed by the proposed topic areas and the questions as they had been set. Additionally, recognising the importance of involving members of the public as active participants rather than the subject of research, a copy of the study protocol – including the interview topic guides – were provided to a local group founded on the principles of INVOLVE (known as the Consumers Research Advisory Group – CRAG), in order to obtain their feedback. The topic guides for the Standard and Telehealth Services covered the person’s views of the service they received; their health and quality of life; how much contact they had with the nurse; and, for those using technology, what they thought of the equipment; whether they considered it to be helpful; and how they reacted to having the technology removed at the end of the eight week programme.

All participants were interviewed in their own home by the same experienced, postgraduate trained qualitative researcher. All of these semi structured interviews took place between June and November 2010 and while taking 25 minutes on average, the interviews lasted between 15 – 45 minutes. Interviews were conducted until sequential analysis identified that information saturation had been achieved, making further interviews unnecessary.

All interviews were audio-taped (following consent), transcribed verbatim and the transcript checked against the recording. The transcripts were then analysed using framework analysis [42] to group the data by individual and by theme. The broad classifications and sub-classifications relevant to the objectives of the study were identified by the research team. Two researchers independently coded all of the patient interviews by intervention (Telehealth or standard service). Coding was marked by highlighter on paper transcripts by each coder, and the two sets of coding were compared and discussed. Very few differences in the coding were noted, and these were typically where two sub-categories applied. The final coding was agreed through discussion with both coders and the interviewer to gain better insight into the context of the answer provided. All highlighted responses and cue questions eliciting those responses were entered against each category and sub-category code and stored in an Excel spreadsheet.

### Patient questionnaires

During the pilot study, recipients of the Telehealth service were invited to complete a semi-structured self-administered questionnaire [see Additional file 2] when the Telehealth equipment was removed at the end of the intervention.

On the first home visit, the staff member from the PCT COPD Service checked the person’s eligibility to participate; explained the study; and provided the patient with a leaflet about the service and a paper copy of the information sheet about the study and a consent form. If the patient agreed to participate in the study the patient was asked to complete two copies of a written consent form. One copy was retained by the clinician and later returned to the research group. The other copy was retained by the participant. Patients who declined to participate in the study, or failed to meet the eligibility criteria [41], received the standard service.

The topics for investigation and the questions posed in the questionnaire were developed based on emergent themes identified through analysis of data from patient interviews undertaken during the feasibility study. As it was hoped that patient data captured during the pilot study could be included with the RCT [41], one of the emergent themes, the demographics of the patient receiving the service, was excluded from the questionnaire as this could remove the anonymity of the patient. Once again, the topics and questions were reviewed by both the research advisory committee and the CRAG prior to submission of the questionnaire to the NHS ethics committee.

Following completion of the intervention, during their final discharge visit, the clinicians provided the recipients of the Telehealth service with a paper copy of the questionnaire. The clinicians identified that researchers were available if the patient required any assistance in completing the form. Seventeen recipients of the Telehealth service completed the self-administered questionnaire, returning them to the research team in the stamped addressed envelopes provided. The questionnaire comprised of open and closed questions covering five aspects of the service: first thoughts on hearing of the service and seeing the equipment; equipment installation and training; use of the equipment; availability of support and removal of the service. Most of the closed questions were augmented by supplementary open questions to explore issues and invite participants to fully disclose their thoughts and perceptions of the service.

The completed questionnaires were coded using the same coding system as for the patient interviews. Two researchers independently coded each of the questionnaires to ensure reliability of coding. Coding was marked on the questionnaires, and all text provided and cue questions eliciting the response entered against each code and stored in an Excel spreadsheet by theme and sub-themes.

### Clinician interviews

All three of the clinicians responsible for delivering the two interventions were interviewed to determine their views of the new service; how technology might help them to deliver a service to patients; and if there had been any issues when selecting some patients to receive the technology from their caseloads when first establishing the new service. For details of the recruitment and consent processes please refer to the ‘ethics approval and consent to participate’ information in the disclosures.

On receipt of the signed consent forms, following a topic guide [see Additional file 3] semi-structured interviews were conducted with the clinicians responsible for delivering both services. As the clinical team delivering the interventions was small, taking time from care delivery to be interviewed had a greater impact on service delivery, so one dyad interview with two clinicians was undertaken and a third clinician was interviewed individually. Both interviews were conducted face-to-face at the clinician’s place of work.

All interviews were audio-taped (following consent), transcribed verbatim and the transcript checked against the recording. The transcripts were then analysed using framework analysis [43] to group the data by individual and by theme, with codes identified inductively from the data. Two researchers independently coded the clinician interviews by intervention (Telehealth or standard service). Coding was marked on the transcripts, and all text provided and cue questions eliciting the response entered against each code and stored in an Excel spreadsheet with sub-themes grouped by overarching themes.

## Results

Analysis of the patient interviews identified seven themes: (1) patient demographics; (2) information received by the participants; (3) installation of the Telehealth technology; (4) Telehealth service functionality; (5) visits; (6) service withdrawal; and (7) service perceptions. The sub-themes that were nested within these overarching themes are shown in Table 3. Responses to the closed questions of the self-administered questionnaire completed by additional recipients of the Telehealth service are summarized in Table 4. Responses to open questions from the questionnaire are incorporated in to the analysis below.

**Table 3:** Analytical themes and sub-theme

Theme		Sub-Theme	
1.0	Patient demographics	1.1	Living arrangements and support
		1.2	Duration of COPD diagnosis
2.0	Information	2.1	How were patients informed about the service
		2.2	Was service enrolment by informed choice
		2.3	Service information provided to the patient
		2.4	Was the service educational regarding their COPD
3.0	Installation of the Telehealth technology	3.1	Was installation straightforward?
		3.2	Who provided instruction on system use?
		3.3	Where the systems were installed in the home
4.0	Telehealth service functionality	4.1	Concern about use of the system
		4.2	Appearance of the system
		4.3	Alerts
		4.4	Incorrect use of the system
		4.5	Simplicity/ease of use of the system
		4.6	Appropriateness of answer categories on the system
		4.7	Embedding the service into daily routine
5.0	Visits	5.1	Number/duration of visits and activities undertaken
		5.2	Number of service clinicians visiting the home
6.0	Service withdrawal		
7.0	Service perceptions	7.1	Feeling of comfort or safety
		7.2	General views of the service
		7.3	Ease of contact with service providers
		7.4	Dislikes
		7.5	Future use of the service
		7.6	Interaction with provider staff
		7.7	View of face-to-face home nursing visits
		7.8	Promotion of the service to others
		7.9	Service integration
		7.10	Could patients tolerate additional questions on their first home visit

**Table 4:**
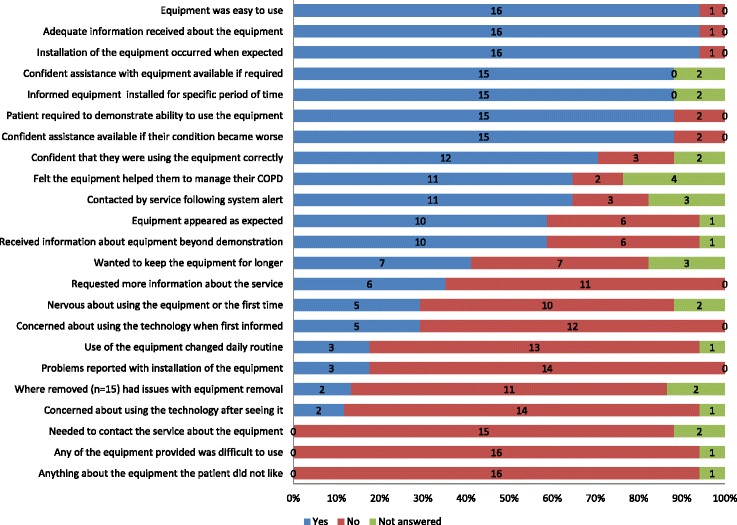
Questionnaire results

### Patient demographics

Five patients who had received the Telehealth supported service were interviewed: three male and two female. Of the three male participants, one lived alone and the other two lived with a spouse, and the two female participants both lived alone. The three participants living alone all reported having a support system of family and neighbours. The time since their COPD diagnosis ranged from eighteen months to over six years. The mean age of recipients of the Telehealth service was 67.22 (SD = 11.60).

Four patients who had recently received the standard service were also interviewed. They were all female; two of whom lived with their spouse and two of whom lived alone. The mean age of recipients of the standard service was 66.59 (SD = 10.54).

As stated previously, as it was hoped that patient data captured during the pilot study could be included with the RCT [41], the demographics of the patients receiving the Telehealth service were excluded from the questionnaire as this could remove the anonymity of the patient.

### Information received by the participants

During the interviews, the patients identified that the decision to accept the Telehealth service when it was offered to them was a conscious, informed choice for most of them. Recipients of the Telehealth service confirmed that they had been provided with the instruction manual, and had used it to find contact telephone numbers, and reviewed information about the technology and the service.

The clinicians who were interviewed noted that their patient cohort was *“quite old and elderly”,* and *“a lot of them are quite frightened by technology”*. On first hearing about using the equipment as part of their care, almost one third of patients completing the questionnaire (*n* = 5) had some concerns and six expressed a degree of nervousness and had asked for more information. Most commonly their questions related to the ease of use of the technology, how the service would work for them, and the use and privacy of their data. Their concerns typically related to their ability to use the equipment correctly, the accuracy of information used for their care, and a lack of familiarity with technology. Being shown the equipment seemed to dispel any concerns, and while the equipment looked as expected to many patients (*n* = 10), to some (*n* = 6) it looked different to how they expected. However, only one patient described it as looking “*a bit technical*”. The clinicians, however, were aware of *“a small minority”* who when *“we show them the unit and how it works and reassure them with questions… (say) ‘oh no, I couldn’t cope with that’”*. Age did not appear to be a barrier to the acceptance of a Telehealth monitoring service or of using the equipment.

Whilst provision of the instruction manual was restricted to recipients of the Telehealth service, recipients of the standard service reported being provided with additional information about COPD and clear guidance on how the eight week intervention would be delivered.

### Installation of the telehealth technology

For both interview (*n* = 4) and questionnaire (*n* = 14) respondents, installation of the Telehealth equipment was generally straight forward, the only exceptions being when telephony service was unavailable creating a brief delay, and a small number of technical issues. The interviews identified that equipment location in the home was dependant on the availability of proximal telephone and power points, although this was not noted as an issue by patients. Whilst service installation staff provided instruction on use of the technology, half of the interviewed patients reported receiving further instruction from the clinicians. The questionnaires revealed that in most instances (*n* = 15) patients were asked to demonstrate that they were able to use the equipment correctly. In one instance where the patient was not asked to do this, the patient themselves identified that they would have preferred this to be done.

### Telehealth service functionality

None of the interviewed patients and only two of the questionnaire respondents identified any initial concerns about using the Telehealth system. After the first use, most patients reported feeling confident in using the equipment. Patients generally (interview *n* = 5, questionnaire *n* = 16) felt the equipment was easy to use and some commented that “*it seemed so easy thought I was doing it wrong”, “it was quite simple to use, there's not a lot to it is there? It’s not rocket science is it?” and “…I was always up and I used to do it at 8’o clock or thereabouts. Only once did I forget it and it was about half past ten when I did it…it became like a routine really and it didn’t take two minutes”.*

Most patients (interview *n* = 4, questionnaire *n* = 11) identified that the system had generated alerts and they had been contacted by telephone by a clinician from the COPD service. Whilst exacerbation of their condition resulted in the majority of the alerts, in their interviews, two patients identified that an alert had been triggered through their pressing the wrong button on the console on one occasion whilst getting used to the equipment. One alert had been triggered by the patient coughing during the night, and the clinician had recommended a change in their medication which alleviated the problem. The standard clinician response to an alert was a telephone call to the patient to confirm current health status or provide advice, faxing a prescription of medication (antibiotics or steroids) to a local pharmacy for timely collection and without the need for a GP visit, and arrangement of a home-visit if deemed necessary.

The patient interviews identified a number of system and service issues. For some of the questions asked on the system, primarily associated with sputum colour or whether the patient was more able to breathe that day, patients noted that the answer categories available to them failed to allow for inter-category answers or for a change in status over the course of the day. To allow clinical staff to review patient data and quickly respond to any alerts patients were required to enter their data before noon each day. With their diminished lung function, preparing for the day is extremely tiring for a COPD patient, and some patients felt that this exertion could elevate their readings. Whilst data submission was identified as taking only a few minutes and easily fitted into the morning routine for most, it was an additional activity to perform each day, and it was also suggested that it may be problematic for those wishing to make other morning appointments. Conversely, others felt that it would be beneficial to be able to have their data checked at any time, given the nature of their condition.

### Visits

In their interviews, for the Telehealth intervention, patients typically identified that one member of the COPD clinical team visited them at home, and the reported visit schedule corresponded with the six identified within the standardised care plan. Most patients receiving the standard service reported receiving visits from three different clinicians, and could not identify who the clinicians were, with one stating that they *“didn’t ask… it didn’t feel right”.*

Patients on the standard service appreciated the education they received from the clinicians regarding their condition, including breathing techniques, and on their medication, such as the use of an inhaler spacer. Whilst the clinicians identified that they provided education to the patients on the Telehealth service, this was not acknowledged by the patients during their interviews.

### Service withdrawal

Patients expressed different emotions regarding the removal of the technology and the conclusion of the Telehealth intervention. In the interviews, whilst some patients were ambivalent about the removal of the equipment, some felt a degree of relief that there was one less activity to perform each morning, and others identified that they liked seeing their readings each day and that they had become used to the system and felt “a little bit lost for a couple of days”.

As their condition had improved, withdrawal of the standard service at the end of the eight week period posed no issues for most of the patients, although some did identify feeling *“a bit deflated”* as the clinician was perceived *“like a fairy godmother with a magic wand, and when she said she was going to sort something, she sorted it”*.

### Service perceptions

Recipients of both services viewed the clinicians as being responsible for keeping them at home, rather than being readmitted to hospital. A feeling of comfort and safety from using the Telehealth equipment was generally derived from patients knowing someone was monitoring their data and knowing “*somebody was looking over me”* and *“keeping an eye on me”;* that *“if anything happened you had somebody there*”; the patients “*knew they were there to help”;* and “*if there was any indication that I was… getting ill… that they’d get in touch”.* One patient stated that they did not *“feel as confident since (the) equipment (was) removed*”. The services were felt to be more integrated, personalised and timely than those received from their GP, with *“problems picked up quickly (and) advice given on how to remedy them*”. In the view of one patient, the standard service had “*cut out all of the jumping through hoop after hoop after hoop and brought it into one place”* and felt it must have “*saved the NHS… thousands of pounds… to be in your own home, and having the proper medication, not having to go anywhere”.*

Having had COPD for an extended period, patients recognised when their condition had worsened, and many could identify when they required medication or hospital intervention to alleviate the symptoms. In prior studies, patients have identified barriers to accessing professional care to manage their condition [27]. Patients consistently reported difficulties when trying to contact their doctor’s practice to make appointments, the lack of appointment availability, and the inability to send someone to collect a prescription on their behalf. Group practices resulted in patients seeing different doctors in successive visits. In their appointment, they felt the doctor they were seeing did not always have current information about their care available, with one reporting that *“he had to ask me what antibiotics I take”*.

Patients reported that their condition often deteriorated while waiting for an appointment to see a doctor, often resulting in a visit to the hospital. Patients also reported receiving many prescriptions, typically of antibiotics or steroids, and frequently for a very short duration, following which their condition deteriorated once again. However, patients identified that they were so familiar with their condition that they challenged what they felt to be inadequate prescribing with one patient noting “*he wanted to give me some steroids… he said right I’ll give you so many, I said no, I have 42… He said I’ll give you them for five days, I said no, I have to have them for seven days… I take six a day for seven days, that’s forty two tablets…”*. They also felt there was a lack of information about medication prescribed for them by their doctors.

Whilst some patients identified that they did not wish for the number of clinician home visits to be further reduced from the level they had experienced, others suggested the clinician visits added little to the functionality of the service as the clinicians asked many of the same questions and took the same readings as the technology. A number of the patients clearly valued the social interaction with the clinician during the home visits.

Recipients of both services reported that it had been clearly identified how patients could contact them for assistance at any time, including out of service delivery hours, with one stating that *“you get anxieties and sometimes you get panicked when you can’t breathe properly and you had a way to get in touch with them that were convenient”*. Patients felt confident that there was someone available if they needed assistance, if their condition deteriorated or if they had issues with the equipment, and this was clearly a valued element of both services.

None of the interviewed patients or questionnaire respondents identified any dislikes with the Telehealth service or the technology. The patients felt it was a *“shame the equipment is not available all the time”* and supported the idea of patients being able to self-manage their condition at home with this type of service. When asked for their thoughts of the Telehealth service, interviewed patients and questionnaire respondents replied with comments such as *“very good”, “couldn’t have asked for more”*, *“(the) service was a success”*, *“the best thing that’s ever come out”*, “*it’s better (service) than your own doctor”* and “*the best (service) I’ve ever had”*.

All of the patients interviewed felt that using the equipment, most specifically taking, and becoming aware of their own, oximetry and blood pressure readings provided them with a greater understanding about their personal health status, with one patient stating “*I felt comfortable that I knew what was happening to myself*”. The clinicians acknowledged that family members often entered data for patients, so the system was providing family, as well as individual education. Most patients felt that the equipment helped them to manage their COPD by allowing them to monitor their physiological data, providing reassurance and decreasing insecurity about their COPD, thereby making them feel more relaxed. The clinicians noted patients *“became more aware of their (oxygen levels), they became more aware of how breathless they actually get in normal activity and what is a daily variance for them. So whereas in the past they’ve just had this chest condition that’s all encompassing they are beginning to focus and find out about what is happening”… “Rather than just thinking ‘I‘m always breathless’, well you know you could say in what context are you always breathless… and it helps them start to understand their condition a lot better”*. The clinicians were receiving more requests to keep the technology, with one asking if they could purchase the system. Several patients confirmed that they wished to keep the equipment for longer, some permanently, with one identifying that they would be following up with the service provider to ensure the return of the system.

## Discussion

A gap in the literature relating to the patient experience of specific Telehealth technologies has been identified [28]. Whilst many studies purport to report on patient perceptions of telehealth systems, in reality they provide findings from satisfaction surveys [45, 46] and are subject to the limitations identified earlier. Where approaches have been used to better elicit patient perceptions [12, 13], these are not reported using a patient perception framework which has resulted in gaps in research and reporting.

In this paper we have presented our findings from semi-structured interviews undertaken with five recipients of the Telehealth Service and contrast their experience with those of four recipients of the standard service. We also reported upon the self-administered questionnaires completed by seventeen recipients of the Telehealth Service. In this section we compare our findings to current knowledge about patient perceptions guided by the thematic areas identified by Larrabee [30]: a) providing for my needs, b) treating me pleasantly, c) caring about me, d) being competent and e) providing prompt care. The quantitative and economic findings from the study have been published previously [41].

The ‘providing for my needs’ dimension includes six concepts: taking care of me, checking on me, responding to my requests, providing pain relief or comfort, giving accurate information and providing a pleasant environment. To date, reporting on this dimension has typically been related to patient satisfaction [12, 45], positive experience with [13], or convenience of [4, 27] the telehealth service, which provides little further information on the attributes contributing to successful service delivery except for elements such as increased [12] or easier [13] contact with providers, [12] or an improved feeling of security [12]. Comparable to earlier studies [23, 25], patients receiving the Telehealth service found it to be beneficial. Our study shows that this benefit is derived even when patients were initially resistant to the concept of technology-enabled care for their condition. As found in other Telehealth studies [24, 25, 27], many patients reported the beneficial feelings of comfort and safety from the Telehealth service. Whilst some studies identified that the availability of physiological readings at home provided this benefit [27], our findings reveal that this reassurance was specifically derived from being able to monitor their physiological readings under clinician supervision. Furthermore, recipients of both services described the ability to contact the clinicians as required, together with the cycle of monitoring, follow-up and issue resolution, as a more integrated service than they had experienced to date, and this is what contributed to their feeling of comfort.

The ‘treating me pleasantly’ dimension refers to communication between the patient and professional. Given the technological nature of Telehealth interventions, it is perhaps not surprising that the human interactions that take place as part of service delivery are typically not included in the evaluation. In the interviews, patients discussed the face-to-face interactions with the service providers and how they perceived “*nothing was too much trouble*” for the clinicians.

The ‘caring about me’ dimension relates to the patient perception that their clinician demonstrates and interest in the individual and cares about them. Like the previous dimension, this speaks to the personal interaction between a clinician and their patient and has typically been overlooked when reporting on studies. Given the demographics of those who are most likely to have COPD and the suggestion that they may be intimidated by technology [24], our study has demonstrated the need for an appropriate recruitment strategy to ensure that people being offered the technology are able to understand and use it confidently. In our study this was achieved by providing an accessible instruction manual and support with training to use the equipment. Additionally, spending adequate time with the patient was required to ensure competency to use the equipment accurately. Examination of data input into the Doc@Home® monitoring system for the 23 patients who completed the Telehealth service during the pilot RCT showed that of the 1175 days of data that should have been entered, patients actually provided input on 1086 days (92.4 %). Patients were able to provide a valid explanation for non-input of data for 51 (4.3 %) of those days. Consequently, only 3.3 % (38 days) of physiological data upload were missing for this patient cohort suggesting that potential barriers to patient adoption can successfully be overcome if adequate care is taken during the development and implementation of a Telehealth monitoring service.

In our study it was interesting to note that for the Telehealth supported service, patients reported being visited by the same clinician for their few visits, but those who received the standard service described being visited by multiple clinicians. Whether this consistency in clinician scheduling had any impact upon the feeling of being ‘cared for’ by patients was not identified and would merit further study in the future.

The ‘being competent’ dimension of good nursing care captures nurses being perceived as using accurate knowledge and skills. Whilst some studies report on the perceived improved efficiency for the nurses [12], there is a gap in reporting on this dimension. Our study showed that patients on the Telehealth service valued the provision of practical solutions to any problems encountered by the patient. One follow-up action most appreciated by the patients involved the clinicians faxing a prescription for medication to a local pharmacy for collection by the patient, or their carer, without the need for a visit with their physician.

The ‘providing prompt care’ dimension is the most reported upon with previous studies frequently identifying, that Telehealth monitoring can assist in the early identification of exacerbations and more timely clinician appointments [27]. Our study showed that patients on the Telehealth service specifically valued the timely telephone contact by the clinicians following a system-generated alert.

It is interesting to note that frequently reported ‘perceptions’ such as familiarity and comfort in using technology [3, 12, 13, 23–25, 27, 45] do not fit with any of the dimensions of this framework of patient perceptions of nursing care. We suggest that this is not a deficiency of the model when considering a technology-enabled intervention, but actually identifies the aspects of nursing care that are truly important to a patient. These dimensions predominantly relate to the human elements of service delivery and can be easily overlooked when developing and implementing a technology-enabled nursing service. However, the importance of these human interactions and activities to the patient cannot be overstated.

Whether the Telehealth intervention could provide a sustainable approach to delivering the service to the potential 1200 patients discharged from the local hospital each year with COPD could not be determined during this study. Whilst the intervention pathway required fewer clinician visits compared to the standard nursing service, more time was required to analyse trends in the physiological data reported for each patient. As clinical staff became more familiar with the technology, they did report that the analytical time per patient was reducing. The registration, implementation and patient education processes became more refined over time, and were not perceived to be a potential barrier to expansion of the service. However, recruitment at a pace comparable to that required for a population of 1200 patients per year was not quite achieved during this study [40] so this could not be confirmed.

## Conclusion

The qualitative findings from an examination of the effectiveness of a Telehealth supported discharge service study suggests that Telehealth home monitoring does have a valuable role to play in the delivery of patient-centred care for those with COPD. However, the introduction of a technology-enabled service of this kind requires extensive planning and a well-supported programme of front line staff education and support to ensure that adequate levels of support are available to patients before they are enrolled, and whilst they are in receipt of the intervention.

This study addresses a current gap in the literature by providing a pragmatic examination of the implementation of a Telehealth monitoring intervention. Although this study was conducted five years ago, the findings are still relevant for health care providers preparing to implement a remote patient telehealth monitoring system as it identifies the patient’s perspectives on informational requirements before being willing to accept a Telehealth monitoring intervention; the installation of the equipment in their home; use of the system, including their view of the specific questions they were required to answer and the time of day they were required to do so; on the number of face-to-face visits received; and their feelings on the withdrawal of the service at the end of the intervention.

By comparing and contrasting the perspectives of patients receiving either a Telehealth monitoring or standard nursing intervention, this study should reassure clinicians that technology can support and engender self-management in patients just as effectively as a more traditional home-visiting nursing service if the patients are provided with the tools and encouragement required.

## Abbreviations

CLAHRC-SYH, Collaboration for Leadership in Applied Health Research and Care for South Yorkshire; COPD, chronic obstructive pulmonary disease; NHS, National Health Service; NIHR, National Institute for Health Research; PCT, primary care trust; RCT, randomised controlled trial

## Additional files

Additional file 1: Patient interview topic guide. (PDF 135 kb) Additional file 2: Patient questionnaire. (PDF 35 kb) Additional file 3: Clinician interview topic guide. (PDF 50 kb)
