# Supplementary material for: Comparison of patient perceptions of Telehealth-supported and specialist nursing interventions for early stage COPD: a qualitative study
Source: BMC Health Serv Res. 2016 Aug 22;16(1):420. doi: 10.1186/s12913-016-1623-z (PMC4994236; doi:10.1186/s12913-016-1623-z)
Supplement: Additional file 1: — Patient interview topic guide. (PDF 135 kb) [file 12913_2016_1623_MOESM1_ESM.pdf]

Pilot study in preparation for a Pragmatic Randomized Controlled Trial of Tele-Health for Early Stage Chronic Obstructive Pulmonary Disease

TOPIC GUIDE

Patients in Technology-Assisted Cohort

1. Aim of Interviews:

To identify why the patient accepted technology as part of their care post hospital discharge for early stage COPD.

2. Introduction:

- Introduce yourself
- Introduce the study i.e. about the participants experience of the technology-assisted community nursing service for COPD
- Reassure re: confidentiality and anonymity:
  - Confirm that the interview is solely for the use of the researchers and any discussions will not be communicated in any way to any service provider
  - The report will pull together findings from all participants in the study and no individual will be identified.
- Remind on length of interview
- Introduce tape recorder and explain transcription, data storage and destruction (post publication of findings)
- Check to see if the participant is willing for the researcher to use a tape recorder
  - If yes, proceed
  - If no, researcher to record notes manually and explain that the written notes are to be used as an aide-memoire for the researcher
- Check if participant has any questions at all at this stage
- Check if participant still happy to participate in the interview
- Thank the person for agreeing to participate

3. Approach:

- Who told you about the technology?
- What were you told about the technology?

- Did they explain everything you needed to know about the technology at that time?
- Did you have any additional questions at that time?
- Were you happy with how everything was explained about the technology?
- Is there anything you would change about your introduction to the technology?

4. First thoughts

- Who told you about the technology that you may be asked to use?
- What was your first reaction?
- Did you have any concerns about the technology?
  - What were they?
    - Did you worry about being able to use it?
    - Did you worry about breaking it?
- Did you ask for any more information about the technology?
  - What did you want to know?
  - Who did you ask?
  - Were they able to answer your questions?
- Were you shown the equipment before you agreed to use it?
- If you were shown the equipment, who showed the equipment to you?
- If you were shown the equipment, what did you think of the equipment when you saw it?
- Did you have any concerns about using the equipment when you saw it?
- How confident were you that there was someone available to help you with the equipment if you needed them?
- Were there any other reasons apart from the ones we have already discussed why you decided that you did want to use any technology as part of your care?

5. Final thoughts:

- Do you have any final thoughts/comments about the equipment?
- Do you have any final thoughts/comments about the service?
- Is there is anything else you would like to ask about the research?
- Thank the participant for their time and contribution
