# Supplementary material for: Comparison of patient perceptions of Telehealth-supported and specialist nursing interventions for early stage COPD: a qualitative study
Source: BMC Health Serv Res. 2016 Aug 22;16(1):420. doi: 10.1186/s12913-016-1623-z (PMC4994236; doi:10.1186/s12913-016-1623-z)
Supplement: Additional file 2: — Patient questionnaire. (PDF 35 kb) [file 12913_2016_1623_MOESM2_ESM.pdf]

The University Of Sheffield.

Pilot study in preparation for a Pragmatic Randomised Controlled Trial of Tele-Health for Early Stage Chronic Obstructive Pulmonary Disease

Patient Questionaire

Community COPD Service Users

Version 1 March 2010

This questionnaire will ask you about the Community COPD Service you have recently received and the technology used to monitor your COPD whilst on the programme. It will provide valuable information when we consider how to provide a service that will have the greatest benefit for people living with COPD.

It will take approximately 20 minutes to complete.

If you need any assistance to complete this questionnaire please do not hesitate to bring this to the attention of your Community COPD nurse or alternatively you can telephone the Barbara Keilly, the project administrator, on (0114 222 1726).

FIRST THOUGHTS:

1. **When you were first told about the technology, were you concerned about using it?** **Yes / No**

   If you answered yes to the above question please answer the following:

   Please explain why you were concerned:

   ____

   ____

   ____

2. **When you were first told about the technology, did you ask for more information about the service?** **Yes / No**

   If you answered yes to the above question please answer the following:

   What did you want to know?

   ____

   ____

   ____

   Who did you ask?______________________________________

   Were they able to answer your questions? **Yes / No**

INSTALLATION OF EQUIPMENT:

3. **Did the installation of the equipment occur when you expected?** **Yes / No**

4. **Were there any problems during the installation?** **Yes / No**

   If you answered yes to the above question please answer the following:

   What were the problems you experienced?

   ____
   ____
   ____

5. **Did the equipment look like you expected?** **Yes / No**

6. **Who taught you how to use the equipment?**

   ____
   ____

7. **How did they teach you how to use the equipment?**

   ____
   ____
   ____
   ____

8. **Did they get you to show them how you would use the equipment?** **Yes / No**

9. **Did you get any other information on how to use the equipment?** **Yes / No**

   If you answered yes to the above question please answer the following:

   What additional information did you receive?

   ____
   ____

10. **Did you feel that you received sufficient information about the equipment?** **Yes / No**

    If you answered no to the above question please answer the following:

    What additional information did you require?

    ____
    ____
    ____

USING THE EQUIPMENT:

11. Did you have any concerns about using the equipment when you first saw it? **Yes / No**

If you answered yes to the above question please answer the following:

What were they?

____

____

____

____

12. Did using the equipment change the way you do anything (e.g. changes to your daily schedule)? **Yes / No**

If you answered yes to the above question please answer the following:

Please explain what changed and why:

____

____

____

13. Was the equipment easy to use? **Yes / No**

14. How often did you use the equipment in an average day?

____

15. Was there anything you found difficult to use? **Yes / No**

If you answered yes to the above question please answer the following:

Could you identify what equipment you found difficult to use any why?

____

____

____

16. Was there anything about the equipment you did not like? **Yes / No**

If you answered yes to the above question please answer the following:

Could you identify what equipment you did not like and explain why?

____

____

____

____

____

17. **Were you nervous about using the equipment for the first time?** **Yes / No**

If you answered yes to the above question please answer the following:

Could you explain why you were worried about using the equipment?

____

____

____

____

18. **Were you confident that you were using everything as you should?** **Yes / No**

19. **How long did it take for you to feel confident in using the equipment?**

    a) **Number of uses:** ___________________________ **<u>OR</u>**

    b) **Number of days/weeks:** ___________________________

20. **Did you feel that the equipment helped you to manage your COPD?** **Yes / No**

If you answered yes to the above question please answer the following:

How did you think the equipment helped you to manage your COPD?

____

____

____

<u>AVAILABILITY OF SUPPORT:</u>

21. **Did you need to contact anyone about the equipment?** **Yes / No**

If you answered yes to the above question please answer the following:

Who did you contact?

____

What did you want to ask them?

____

____

____

Did they answer your question? **Yes / No**

22. **Were you confident that there was someone available to help you with the equipment if you needed them?** **Yes / No**

23. Did anyone from the Community COPD Service contact you to say that the system had alerted them that you may need assistance? **Yes / No**

If you answered yes to the above question please answer the following:

Who contacted you? ____

How did they contact you?____

What did they do?____

How did you feel about them contacting you?

____

____

24. Were you confident that there was someone to help you if your condition became worse? **Yes / No**

REMOVAL OF THE EQUIPMENT:

25. Were you told that the equipment would be installed for a specific amount of time? **Yes / No**

26. How did you feel when you were told that you would only have the equipment for a specific period of time?

____

____

____

27. Did you want to keep the equipment for longer?

If you answered yes to the above question please answer the following:

Can you explain why?

____

____

____

28. Were there any problems with the removal of the equipment? **Yes / No**

If you answered yes to the above question please answer the following:

If there were any problems could you please explain what they were:

____

____

____

29. Do you have any final thoughts or comments about the equipment that you would like to share?

30. Do you have any final thoughts or comments about the service that you would like to share?

Thank you for taking the time to complete this survey.
