# Supplementary material for: Comparison of patient perceptions of Telehealth-supported and specialist nursing interventions for early stage COPD: a qualitative study
Source: BMC Health Serv Res. 2016 Aug 22;16(1):420. doi: 10.1186/s12913-016-1623-z (PMC4994236; doi:10.1186/s12913-016-1623-z)
Supplement: Additional file 3: — Clinician interview topic guide. (PDF 50 kb) [file 12913_2016_1623_MOESM3_ESM.doc]

**
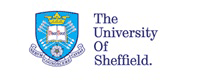
**

**Modelling study in preparation for a Pragmatic Randomized Controlled Trial of Tele-Health for Early Stage Chronic Obstructive Pulmonary Disease**

**TOPIC GUIDE**

***Focus group with nursing staff of SDS***

# Aim of Focus Groups:

To examine the views of the specialsist nursing staff of the new SDS and technology-assisted SDS for patients with early stage COPD.

# Introduction:

- Introduce yourself
- Introduce the study i.e. about the participants experience of the technology-assisted community nursing service for COPD
- Reassure re: confidentiality and anonymity:
  - Confirm that the focus group is solely for the use of the researchers and any discussions will not be communicated in any way to any service provider
  - The report will pull together findings from all participants in the study and no individual will be identified.
- Remind on length of focus group
- Introduce tape recorder and explain transcription, data storage and destruction (post publication of findings)
- Check if group members have any questions at all at this stage
- Check if group members still happy to participate in the focus group
- Thank the group for agreeing to participate

# First thoughts

- What did you think when you were first told about the standard SDS programme?
- What did you think when you were first told about the technology-supported SDS programme?
- Did you have any concerns about either of the new services?
  - What were they?
- Did you ask for any more information about the new services?
  - What did you want to know?
  - Who did you ask?
  - Were they able to answer your questions?

**FOR THE STANDARD SDS:**

# Recruitment:

- Do you feel that all patients that could benefit are being referred to this service?
  - If not, why not?
  - How could this be improved?

# First Visit:

- How soon after discharge from the hospital do you do your first visit for both programmes?
  - What do you do during that visit?
  - What information do you collect?
  - What do you tell them about the SDS programme?

# Second Visit:

- When does this visit take place?
  - What do you do during that visit?
  - What information do you collect?

# Third Visit:

- When does this visit take place?
  - What do you do during that visit?
  - What information do you collect?

# Fourth Visit:

- When does this visit take place?
  - What do you do during that visit?
  - What information do you collect?

# Additional Visits:

- Is there a need for additional (unplanned) visits?
  - What do you typically do during these visits?
- How do you feel that this standard SDS service is working?
  - Is there anything you would do differently? If so what and how?
  - Are there any problems?

**FOR THE TELEHEALTH SUPPORTED SDS:**

# Recruitment:

- Do you feel that all patients that could benefit are being referred to this service?
  - If not, why not?
  - How could this be improved?
- Do you feel that the service is being offered to patients inappropriately?
  - How could this be addressed?

# First Visit:

- How soon after discharge from the hospital do you do your first visit for both programmes?
  - What do you do during that visit?
  - What information do you collect?
  - What do you tell them about the SDS programme?
  - What do you tell patients about the technology?
  - When is the patient told that the equipment will be installed?
  - Are they told who will install it?
  - Are they told who will train them to use it?
  - Are they told who to contact if they have problems?
  - Are they given the option to stay with the Standard Discharge System?
    - Do any choose to do so?
    - If yes, why?
    - What do you do in this situation? (Next steps?)

# Second Visit:

- When does this visit take place?
  - What do you do during that visit?
  - What information do you collect?

# Third Visit:

- When does this visit take place?
  - What do you do during that visit?
  - What information do you collect?

# Additional Visits:

- Is there a need for additional (unplanned) visits?
  - How are these visits initiated (in the area/patient telephone call)?
  - What do you typically do during these visits?

# Installation of Equipment:

- Does the installation of the equipment always occur when you expect?
- Have there been any problems during the installation that you are aware of?
  - What were they?
  - How were they resolved (assuming that they were)?
  - How could they be prevented in the future

# Equipment Training:

- Who taught you how to use the equipment?
- How did they teach you how to use the equipment?
- Did you get any other information on how to use the equipment?
- Did they explain everything you needed to know about the equipment?

# Experience of the equipment:

- What did you think about the equipment when you first saw it?
- Did you have any concerns about the ability of patients to use the equipment when you first saw it?
- What are your thoughts now?
  - Is it your experience that patients are comfortable using the equipment?
  - Have patients had problems with the equipment at all?
    - What were they?
    - How were they resolved?
    - By whom?
- Does using the equipment create more/less work for you? Please explain.

# Acceptability of the Equipment:

- How do you feel about patients using the equipment?
- How do you feel about the equipment?
- Have there been any problems with the patients using the equipment?
  - Technical – e.g. do they understand it?
  - Practical – e.g. do they use it as often as they should?
  - Accidental – e.g. trip/falls over the leads?
- Were others (carers, family members, partners) comfortable with the patients using it?
- Were you confident that patients use everything as they should?
- Do you feel that the equipment helps patients to manage their COPD?
- How do you think the equipment helps patients to manage their COPD?
- Do you think the technology allows you to deliver a different type of service?
  - What are the differences?
  - Do you think you can offer a better service or does it detract from the service provided? Please explain.

# Availability of Support:

- How many times do you have to go out to visit patients who are having problems with the equipment?
- Have you had to contact anyone about the equipment?
  - Who did you contact?
  - How did they help you?

# Removal of the equipment

- How do patients react to being told they will only have the equipment for 8 weeks?
- How do they react when the equipment is taken away?

# Review

- Is there anything about the SDS and technology-supported SDS that you would change?
- Do all the stages of both services flow as you expect or are there any gaps or problems?

# Final thoughts:

- Do you have any final thoughts/comments about the services as they are currently provided?
- Do you have any final thoughts/comments about the telehealth equipment?
- Is there is anything else you would like to ask about the research?
- Thank the group members for their time and contribution
